# An Update Evolving View of Copy Number Variations in Autoimmune Diseases

**DOI:** 10.3389/fgene.2021.794348

**Published:** 2022-01-20

**Authors:** Rong-hua Song, Chao-qun Gao, Jing Zhao, Jin-an Zhang

**Affiliations:** Department of Endocrinology and Rheumatology, Shanghai University of Medicine and Health Sciences Affiliated Zhoupu Hospital, Shanghai, China

**Keywords:** copy number variations, single-nucleotide polymorphism, autoimmune disease, autoimmune thyroid disease, systemic lupus erythematosus

## Abstract

Autoimmune diseases (AIDs) usually share possible common mechanisms, i.e., a defect in the immune tolerance exists due to diverse causes from central and peripheral tolerance mechanisms. Some genetic variations including copy number variations (CNVs) are known to link to several AIDs and are of importance in the susceptibility to AIDs and the potential therapeutic responses to medicines. As an important source of genetic variants, DNA CNVs have been shown to be very common in AIDs, implying these AIDs may possess possible common mechanisms. In addition, some CNVs are differently distributed in various diseases in different ethnic populations, suggesting that AIDs may have their own different phenotypes and different genetic and/or environmental backgrounds among diverse populations. Due to the continuous advancement in genotyping technology, such as high-throughput whole-genome sequencing method, more susceptible variants have been found. Moreover, further replication studies should be conducted to confirm the results of studies with different ethnic cohorts and independent populations. In this review, we aim to summarize the most relevant data that emerged in the past few decades on the relationship of CNVs and AIDs and gain some new insights into the issue.

## Introduction

Copy number variations (CNVs), as a main type of structure variation (SV) caused by genomic rearrangement, mainly include deletion and duplication of sub-microscopic but large genomic segments ranging from 1 kb to 3 Mb (Redon et al., 2006). Single-nucleotide polymorphisms (SNPs) have been recognized to be involved in many autoimmune diseases (AIDs) (Song et al., 2021a; Song et al., 2021b; Jiang et al., 2021); however, CNVs containing more nucleotide content per genome than SNPs are responsible for a large proportion of human genetic variation and show an importance in genetic diversity and evolution (Redon et al., 2006; Cleynen et al., 2016). Thus, more attention has been paid to the research of CNVs in diseases. Nowadays, the genome-wide assays for CNV study include array-based comparative genomic hybridization (aCGH), SNP genotyping microarrays, next-generation sequencing, and long-read sequencing techniques (Hehir-Kwa et al., 2018). There are several main categories in the molecular mechanisms during the process of CNV formation, DNA recombination, rearrangement, and error replication. Besides, CNVs also have several types, such as insertions, deletions, inversions, and translocations (Feuk et al., 2006; Human Genome Structural Variation Working Group et al., 2007). Numerous reports imply that CNV is one of the main genetic factors underlying human diseases, including AIDs (Hauptmann et al., 1974; Tomer and Davies, 2003; Iafrate et al., 2004; Mack et al., 2004; Redon et al., 2006; Yang et al., 2007; Yim et al., 2010). The mechanisms underlying the involvement of CNVs in clinical phenotypes are mainly gene disruption and rearrangement (McCarroll and Altshuler, 2007; Yim et al., 2010). Further deep studies on CNVs have shed new light on human genome structure, genetic variations between individuals, and genetic pathogenic factors of human AIDs.

AIDs usually share possible common mechanisms, i.e., a defect in the immune tolerance exists due to diverse causes from central and peripheral tolerance mechanisms. Although majority of human genetic variations do not contribute to overt diseases (Tomer and Davies, 2003), some genetic variations including SNPs, nucleotide insertions/deletions, structural variations, and CNVs are known to link to several AIDs during the past few decades and are of importance in the susceptibility to AIDs and the potential therapeutic responses to medicines (Iafrate et al., 2004; Redon et al., 2006). Studies have revealed that some SNPs are related to AIDs and could be genetic mechanisms underlying the development of AIDs (Song et al., 2021a; Song et al., 2021b; Jiang et al., 2021). Structural variations including complex rearrangement of segments with sizes of thousands to millions of base pairs have been recognized as a rich source of genetic diversity. CNVs as a crucial source of genomic diversity caused by the rearrangement of genome are ubiquitously presented in human genome and may affect the susceptibility to many diseases (Iafrate et al., 2004). During the past years, thousands of gene CNVs have been reported (Redon et al., 2006). However, most studies focus on their relationship with tumors and chronic diseases (Jia et al., 2011; Saadati et al., 2016; Voll et al., 2017). Moreover, CNVs are very common in genomic regions encoding immune-related genes, which are closely related to the etiology of AIDs. Thus, they potentially impact polygenic autoimmunity and may lead to the imbalance of the autoimmune system and the development of some AIDs. Additionally, some common CNVs have also been reported to be correlated to several specific AIDs (Mamtani et al., 2008; Mamtani et al., 2010; Liu et al., 2011). Yim et al. (2015) reviewed studies on the clinical implications of copy number variations in autoimmune disorders in detail. However, the relationship between CNVs and the pathogenesis of AIDs has not been fully revealed. Furthermore, in recent years, as new technologies develop, researches on CNVs have made new discoveries and progress. Herein, based on the emergence of numerous studies on the relationship between CNVs and AIDs in the past decades, we reviewed all the related studies and summarized their findings in order to provide new ideas for future explorations and to uncover the mechanisms underlying AIDs (Table 1).

**TABLE 1:** Copy number variant loci or genes related to autoimmune diseases.

CNV-related genes or regions	Autoimmune diseases/syndromes	Populations (sample size)	CNV detection methods	Most common copies in healthy normal controls	Risk-associated CNVs (*p*-values)	Results	References
*Beta-defensin gene*	Psoriasis	Dutch and German population (179 Dutch patients and 272 controls; 319 German patients and 305 controls)	High-throughput paralogue ratio test (PRT)	40% controls were four copies	Higher gnomic copy number (*p* = 0.01 for Dutch, *p* = 7.8E−5 for German)	Consistent	Hollox et al. (2008)
*BMP8A*	AS	Iranian (450 patients and 450 healthy controls)	TaqMan real‐time polymerase chain reaction (PCR)	Two copies	No significant association	–	Shahba et al. (2018)
*C3* and *C5*	BD	Han Chinese population (1,064 patients and 2,174 controls)	Real-time PCR	Two copies of C3 and C5	More than two copies of C3 (*p* = 5.5E−3) and C5 (*p* = 1.1E−8)	–	Xu et al. (2015)
*C4* and its two isotypes, *C4A* and *C4B*	SLE	Caucasians (Columbus): 1,241 European Americans (Yang et al. 2007) and Brazilian (427 patients and 301 controls)	TaqI southern blots (Yang et al. 2007); real-time PCR (Pereira et al. 2019)	Four copies of C4 genes (majority)	Lower copy number of C4 and C4A (*p* = 0.00002; Yang et al. 2007); low total copy number of C4 (*p* < 0.001), C4A (*p* < 0.001), and C4B (*p* = 0.03)	Consistent	Hauptmann et al. (1974), Yang et al. (2007), and Pereira et al. (2019)
		221 Caucasians (North American)	TaqMan-based real-time PCR and Southern blotting	Not reported	Higher copy number of C4B associated with hypertension and effective response to statin therapy in childhood-onset SLE patients (*p* = 0.016) and higher diastolic blood pressure (*p* = 0.015)	–	Mulvihill et al. (2019)
	CD	Belgian population (770 patients and 345 controls)	Array comparative genomic hybridization	Not reported	Lower C4L (*p* = 7.68E−3) and higher C4S copies (*p* = 6.29E−3)	–	Cleynen et al. (2016)
	BD	Han Chinese population (905 patients and 1,238 controls)	Real-time PCR	Four copies of C4 genes, 2 copies of C4A genes	More than two copies of C4A (*p* = 1.65E−7)	–	Hou et al. (2013)
	T1DM	American population (cohort 1: 50 patients and 57 controls; cohort 2: 110 patients)	TaqMan quantitative PCR	Four copies of C4 genes; two copies of C4A gene	Fewer copies of C4A (*p* = 1.76E−5) and HERV-K (C4) (*p* = 4.59E−7)	–	Mason et al. (2014)
	GD	Chinese population (624 patients and 160 healthy individuals)	Quantitative real-time polymerase chain reaction	Four copies of C4 genes; two copies of C4A and C4B genes	Four copies of C4 (*p *= 0.001); copies of C4A (*p* = 0.008) and C4B (*p* = 2.42E-5) in GD	–	Liu et al. (2011)
*CCL3L1*	SLE	Caucasians (San Antonio), 1,084 subjects (469 cases of SLE and 615 matched controls)	59-RACE and reverse transcription-PCR (RT-PCR)	Two copies	Lower than or greater than two copies (*p* = 0.032)	–	Mamtani et al. (2008)
	RA	Caucasian (1,136 patients and 1,470 controls; McKinneyet al.2008), Tunisians and French (100 French patients and 200 controls; 166 Tunisian patients and 102 controls; Ben Kilani et al. 2016), United Kingdom population (657 patients and 192 controls), and United Kingdom (274 patients and 276 controls)	Reverse transcriptase (RT)-PCR (McKinney et al. 2008), droplet digital PCR (ddPCR) (Ben Kilani et al. 2016), PRT methodology (Carpenter et al. 2011)	Two copies	Higher than two in the New Zealand cohort (*p* = 0.009) but not the United Kingdom cohort; no association in the French study, a protective effect of five copies in the Tunisian population (*p* value not reported (Ben Kilani et al. 2016), no association (Carpenter et al. 2011)	Controversial	McKinney et al. (2008), Ben Kilani et al. (2016), and Carpenter et al. (2011)
	CD and psoriasis	United Kingdom (657 CD patients, 202 psoriasis patients, and 276 controls)	PRT methodology	Not reported	No association	–	Carpenter et al. (2011)
	AS	Algerian (81 patients and 119 controls)	Digital droplet PCR (ddPCR)	Two copies	No association	–	Dahmani et al. (2019)
	T1DM	Caucasian population (252 patients and 1,470 controls; McKinney et al. 2008) and 2,000 patients and 3,000 controls (Wellcome Trust Case Control Consortium et al. 2010)	Reverse transcriptase (RT)-PCR (McKinney et al. 2008) and the Agilent Comparative Genomic Hybridization (CGH) platform (Wellcome Trust Case Control Consortium et al. 2010)	Two copies	A copy number higher than 2 (*p* = 0.064) in the Caucasian population (McKinney et al. 2008); no association in the study (Wellcome Trust Case Control Consortium et al. 2010)	Consistent	McKinney et al. (2008) and Wellcome Trust Case Control Consortium et al. (2010)
*CD40*, *PTPN22*, *and CTLA-4*	GD	Caucasian (191 patients and 192 controls)	Quantitative-PCR (Q-PCR) assays	Two copies	No copy variation in the CD40, CTLA-4, and PTPN22 gene number variation in GD	–	Huber et al. (2011)
*CFH*, *CFHR1*, *KIAA0125*, *UGT2B15*, *UGT2B17*, *TRY6*, and *CCL3L1*	GD	Chinese Han population (144 patients and 144 controls)	TaqMan quantitative polymerase chain reaction (TaqMan qPCR)	Two copies	No association	–	Song et al. (2017)
*DEFA1*	BD	Korean population (55 patients and 35 controls)	A duplex TaqMan^®^ real-time PCR assay	Most samples (31.1%) had a CN of five	A high CN associated with intestinal involvement in BD patients (*p* = 0.005)	–	Ahn et al. (2012)
*DEFB4*	BD	Korean population (197 patients and 197 controls)	A novel comparative multiplex polymerase chain reaction (PCR): paralogue ratio test (PRT)	Median copy number was four	Lower copy number, but no statistical difference (*p* = 0.245)	–	Park et al. (2011)
*DEFA1A3*	CD	Danes (240 patients)	Combined real-time quantitative PCR and pyrosequencing	Mean copy number was 6.7	Higher copy number (*p *< 0.001)	–	Jespersgaard et al. (2011)
*DEFB103*	AS	Chinese population (406 patients and 401 controls)	A multiplex fluorescence competitive polymerase chain reaction (PCR)	Ranged from 2 to 6	No association	–	Cai et al. (2015)
*FAS, caspase8, caspase3*, and *BCL2*	BD	Han Chinese population (1,014 patients and 2,076 controls)	TaqMan copy number assays and real-time PCR	Diploid 2 copy number carriers	High FAS copy number (>2) (*p* = 1.05E−3 in the first-stage study, *p* = 3.35E−8 in the replication and combined study)	–	Yu et al. (2015)
*FCGR3A*	SLE	Taiwanese population (846 patients with SLE and 1,420 healthy control subjects)	Custom TaqMan CNV real-time quantitative polymerase chain reaction (PCR) assays	Two copies	A low FCGR3A copy number (<2) (*p *= 5.06E−4) and a high (>2) FCGR3A copy number (*p *= 0.003)	–	Chen et al. (2014)
	RA	Taiwanese population (948 patients with RA and 1,420 healthy control subjects)	Custom TaqMan CNV real-time quantitative polymerase chain reaction (PCR) assays	Two copies	A low copy number (*p* = 5.83E−4)	–	Chen et al. (2014)
	AS	Algerian (81 patients and 119 controls; Dahmani et al.2019), Chinese population (402 patients and 399 controls; Wang et al.2016)	Digital droplet PCR (ddPCR) (Dahmani et al. 2019) and AccuCopy™ method (Wang et al. 2016)	Two copies	Less than two copies (<2) (*p* = 0.0001; Dahmani et al. 2019), a low copy number (*p *< 0.001; Wang et al. 2016)	Consistent	Dahmani et al. (2019) and Wang et al. (2016)
*FCGR3B*	SLE	United Kingdom Caucasians (171 patients and 176 controls; Willcocks et al. 2008), Spanish ancestry (146 patients and 409 controls; Mamtani et al. 2010), Afro-Caribbean (134 patients and 589 controls; Molokhia et al. 2011), Taiwanese (846 patients and 1,420 controls; Chen et al. 2014), and Brazilian population (135 unrelated SLE patients and 200 healthy unrelated subjects; Barbosa et al. 2018)	qPCR (Willcocks et al. 2008), real-time PCR (Mamtani et al. 2010), paralogue ratio test (PRT) assay (Molokhia et al. 2011), custom TaqMan CNV real-time quantitative polymerase chain reaction (PCR) assays (Chen et al. 2014), and quantitative real-time PCR (Barbosa et al. 2018)	Two copies	A lower (<2) copy number in United Kingdom Caucasians (*p* = 0.027; Willcocks et al. 2008), copy number <2 or >2 in cases of Spanish ancestry (*p* = 0.001 and 0.013, respectively; Mamtani et al. 2010), Afro-Caribbean (*p* = 0.02; Molokhia et al. 2011), Taiwanese (*p* = 0.0032; Chen et al. 2014), and Brazilian population (*p* = 1.66E−3; Barbosa et al. 2018)	Consistent	Willcocks et al. (2008), Mamtani et al. (2010), Molokhia et al. (2011), Chen et al. (2014), and Barbosa et al. (2018)
	RA	Spanish ancestry (158 patients and 409 controls; Mamtani et al. 2010), South Australia (197 patients and 162 controls; Graf et al. 2012), Taiwanese (948 patients and 1,420 controls; Chen et al. 2014), British population (480 patients; Rahbari et al. 2017), French population (Bai Kilani et al. 2019)	Real-time PCR (Mamtani et al. 2010), custom TaqMan^®^ CN assay (Graf et al. 2012), custom TaqMan CNV real-time quantitative polymerase chain reaction (PCR) assays (Chen et al. 2014), a PRT/REDVR approach (Rahbari et al. 2017), and droplet digital PCR (Bai Kilani et al. 2019)	Two copies	No association in cases of Spanish ancestry (Mamtani et al. 2010), lower and higher copy number in South Australia (*p* = 0.017; Graf et al. 2012), no association in Taiwanese (Chen et al. 2014), deletion in British population (*p* = 2.9E−3; Rahbari et al. 2017), and without null allele (one–three copy numbers) in French population (Bai Kilani et al. 2019)	Controversial	Mamtani et al. (2010), Graf et al. (2012), Chen et al. (2014), Rahbari et al. (2017), and Bai Kilani et al. (2019)
	UC	Japanese population (752 patients and 2,062 controls)	TaqMan assay	Not reported	Abnormal copies (*p* = 0.02)	–	Asano et al. (2013)
	Psoriasis	Han Chinese population (343 patients and 574 controls)	TaqMan^®^ copy number assays	Not reported	A higher copy number (*p *< 0.02)	–	Wu et al. (2014)
	BD	Iran (187 patients and 178 controls)	Quantitative real-time PCR	Two copies	No association		Black et al. (2012)
	AS	Algerian (81 patients and 119 controls; Dahmani et al. 2019) and Chinese population (402 patients and 399 controls; Wang et al. 2016)	Digital droplet PCR (ddPCR) (Dahmani et al. 2019) and AccuCopy™ method (Wang et al. 2016)	Two copies	No association in Algerian population (Dahmani et al. 2019) and a low (≤3) FCGR3B copy number (*p* = 0.001; Wang et al. 2016)	Controversial	Dahmani et al. (2019) and Wang et al. (2016)
	pSS	Spanish ancestry (61 patients and 409 controls), Australian (174 patients and 162 controls; Nossent et al. 2012), and Swedish and Norwegian population (124 patients and 139 controls; Haldorsen et al. 2013)	Real-time PCR (Mamtani et al. 2010), a quantitative real-time polymerase chain reaction assay (Nossent et al. 2012), TaqMan copy number assay (Haldorsen et al. 2013)	Two copies	Copy number <2 or >2 in cases of Spanish ancestry (*p* = 0.074 and 0.048, respectively; Mamtani et al. 2010), low FCGR3B CN (<2 copies) in Australian population (*p* = 0.016; Nossent et al. 2012), and no association in Swedish and Norwegian population Haldorsen et al. (2013)	Controversial	Mamtani et al. (2010), Nossent et al. (2012), and Haldorsen et al. (2013)
*GPC5*, *B9D2,* and *ASB11*	AITD	Chinese Han population (158 patients and 181 controls)	Chromosome microarray on the Affymetrix CytoScan™ HD platform, then identified by RTPCR	Not reported	The frequency of CNV loss for GPC5, B9D2, and ASB11 genes was higher in AITD (*p *< 0.05)	–	Guan et al. (2020)
*GSTM1*	RA	Swedish (2,426 cases and 1,257 controls) and Tunisian population (165 cases and 102 controls)	TaqMan copy number assays (Lundstrom et al. 2011) and digital droplet PCR (ddPCR) (Achour et al. 2018)	51.8% for 0 copies and 40.1% for 1 copy	No association in Swedish population (Lundstrom et al. 2011) and lack of association (Achour et al. 2018)	Consistent	Lundstrom et al. (2011) and Achour et al. (2018)
*HBD-2*	CD	German	Genome-wide DNA copy number profiling by array-based comparative genomic hybridization and quantitative polymerase-chain reaction analysis	Median of 4 (range 2–10) copies	The copy number distribution shifted to lower numbers (*p* = 0.002)	–	Fellermann et al. (2006)
*HSP90* and its two major isoforms	SLE	Han Chinese population (419 patients and 538 controls)	A custom-by-design Multiplex AccuCopy™ method	Two copies	Abnormal copies of HSP90AB1 (*p* = 0.02)	–	Zhang et al. (2019)
*KIR3DL1* and *KIR3DS1*	T1DM	White European ancestry (6,744 cases and 5,362 controls)	A hybrid qPCR/SNP array	Two copies of KIR3DL1 and 0 copy of KIR3DS1	No evidence of association	–	Pontikos et al. (2014)
*LCE3B* and *LCE3C*	Psoriasis	The Dutch population (1,039 cases and 759 controls)	Array comparative genomic hybridization and a polymerase chain reaction: TaqMan SNP genotyping assay	Not reported	Absence (*p *< 0.0001)		Bergboer et al. (2012)
*IL17F*, *IL23A*, *IL17A*, and *IL23R*	BD	Han Chinese population (1,036 patients and 2,050 controls)	TaqMan real-time polymerase chain reaction assay	Not reported	More than two copies of IL17F (*p* = 4.17E−8) and IL23A (*p* = 2.86E−11) associated with BD and no association between CNV of IL17A and IL23R	–	Hou et al. (2015)
*IL-22 gene exon1*	Psoriasis	Estonian (290 patients and 263 controls)	Quantitative RT-PCR	Not reported	Abnormal copies associated with psoriasis severity (*p *< 0.0001)	–	Prans et al. (2013)
*miR-143*, *miR-146a*, *miR-9-3*, *miR-205*, *miR-301a*, and *miR-23a*	AS	Chinese Han population (768 patients and 660 controls)	TaqMan PCR	Two copies	Low copy numbers of miR-143 (*p* = 1.126E−7), miR-146a (*p* = 3.716E−8), miR-9-3 (*p* = 2.566E−5), and miR-205 (*p* = 7.187E−6) and high copy numbers of miR-301a (*p* = 3.725E−5) and miR-23a (*p* = 8.033E−9) AAU+, AS+; additionally, a low copy number of miR-146a (*p* = 0.001) and a high copy number of miR-23a and miR-205 (*p* = 0.002) in AAU+, AS−	–	Yang et al. (2017)
*miR-23a, miR-146a*, and *miR-301a*	BD	Han Chinese population (377 patients and 2,291 controls)	TaqMan PCR	Two copies	No association	–	Hou et al. (2016)
*NCF1*	RA	The middle and southern parts of Sweden (494 patients and 480 controls)	A multiplex qPCR assay	Two copies	RA less likely to have an increased copy number (*p* = 0.037)	–	Olsson et al. (2012)
*SIRPB1* and *TMEM91*	AITD	Chinese Han population (15 patients and 15 controls)	Chromosome microarray	Not reported	The frequency of CNV gain for SIRPB1 was higher in AITD (*p* = 0.001) and no association of TMEM91 CNV and AITD	–	Jin et al. (2018)
*T-Bet*, *GATA-3*, *RORC*, and *FOXP3*	AS	Chinese Han population (676 patients with AAU, including 298 patients with AAU+, AS+; 378 patients with AAU+, AS−; and 596 unrelated healthy controls)	Real-time PCR	Two copies	A high copy number (CN) of T-bet in AAU+, AS− and AAU+, AS+ (*p* = 4.3E−5 and 1.2E−8, respectively), a high CN of GATA-3 in AAU+, AS+ (*p* = 1.8E−7), a higher frequency of CN of FOXP3 in female AAU+, AS+ and female AAU+, AS− (*p* = 0.005 and 0.004, respectively), and no association between RORC CNVs and AS	–	Bai et al. (2016)
*TBX21*, *GATA3*, *Rorc*, and *Foxp3*	BD	Han Chinese population (1,048 patients and 2,236 controls)	TaqMan real-time PCR	Two copies of these four genes	High Rorc CNV in BD (*p* = 8.99E−8) and low Foxp3 CNV (*p* = 1.92E−5) in female BD	–	Liao et al. (2015)
*TLR7*	BD	Chinese Han population (400 patients and 600 controls)	Real-time PCR	One copy for male and two copies for female	A high copy number of TLR7 (*p* = 0.021 for males and *p* = 0.048 for females)	–	Fang et al. (2015)
	AS	Chinese Han population (649 patients and 628 controls)	AccuCopy^TM^ method	Not reported	Lower copy number (=1), especially in males (*p* = 0.009 for TLR7_1 fragment and *p* = 0.01 for TLR7_2 fragment)	–	Wang et al. (2018)
	GD and GO	Chinese population (196 controls and 484 GD patients, including 203 patients with GO)	Real-time polymerase chain reaction (PCR)	Not reported	A protective effect of lower than normal CNV for TLR7 (CNV <2 for females and CNV <1 for males) but no statistical significance and no association in GO	–	Liao et al. (2014)
*TSHR*	GD and GO	Chinese population (196 controls and 484 GD patients, including 203 patients with GO)	Real-time polymerase chain reaction (PCR)	Two copies	Copy number <2 or >2 in GD, not in GO (*p* = 0.01)	–	Liao et al. (2014)
*UGT2B17*	AS	Newfoundland (298 patients and 299 controls)	Built-in DNA analytics aberration detection method-2 (ADM-2) algorithm	Two copies	The frequency of two copies higher in cases (*p *< 0.05)	–	Uddin et al. (2013)

CNVs, copy number variations; SLE, systemic lupus erythematosus; RA, rheumatoid arthritis; IBD, inflammatory bowel disease; BD, Behcet’s disease, AS, ankylosing spondylitis (AS); pSS, primary Sjogren’s syndrome; T1DM, type 1 diabetes mellitus; AITD, autoimmune thyroid disease

## Systemic Lupus Erythematosus

Systemic lupus erythematosus (SLE), with a prevalence rate of eight–nine times higher in females than in males during childbearing age, is a typical systemic autoimmune inflammatory disease with a strong genetic susceptibility and is characterized by the production of autoantibodies and the existence of chronic inflammation. There is a wide range of clinical features in SLE patients, such as discoid lesions, nephritis, arthritis, and malar rash (Barbosa et al., 2018). So far, the exact genetic physiology of SLE remains an open question. Complement is well known to be involved in many immune-mediated diseases. Among them, complement component C4 is a pivotal effector of the immune system. There are two common isoforms of C4: C4A and C4B, and 95% of C4A and 54% of C4B contain an endogenous retroviral sequence in their ninth intron, HERV-K (C4) (Wouters et al., 2009). HERV-K (C4) can cause the antisense transcription of C4 as it is oriented opposite to C4 (Mack et al., 2004). According to the presence or absence of HERV-K (C4), there are two different size varieties of isotypes for both C4A and C4B: C4L (long) and C4S (short) (Mack et al., 2004; Wu et al., 2008). Hauptmann et al. (1974) first found the deficiency of *C4* copy number in SLE patients. Later, Yang et al. (2007) uncovered that SLE patients had lower copy number of total *C4* and *C4A* genes than healthy volunteers. Pereira et al. (2019) observed that the risk of developing SLE was 2.62 times higher in subjects with low total *C4* copy number and 3.59 times higher in subjects with low *C4A* copy number. These consistent results imply that deletion or deficiency of *C4* or *C4* isotypes will increase the risk for SLE. The potential mechanism is like this, the decrease of *C4* CNVs will cause C4 deficiency, bring the impairment of autoantigene clearance and the negative selection of auto-reactive B cells, and then favor the onset of SLE (Pereira et al., 2019). In addition, there was another study aiming at the relationship between *C4* CNVs and the drug response to treatment of SLE. The study of Mulvihill et al. (2019) found that higher copy number of complement *C4B* and elevated serum complement levels were associated with hypertension and effective response to statin therapy in childhood-onset SLE patients.

C-C chemokine ligand 3 like-1 (CCL3L1) is a potent ligand for the HIV coreceptor, and C-C chemokine receptor 5 (CCR5) is an important factor in immune response (Gonzalez et al., 2005). Mamtani et al. (2008) found that the CNVs of *CCL3L1–CCR5* were strong predictors for the overall risk of SLE and high autoantibody titers and lupus nephritis, and subjects with lupus nephritis differentially recruit leukocytes.

Receptors for the Fc portion of IgG are involved in the handling and clearance of immune complex and in the regulation of B cell activation during SLE development (Niederer et al., 2010). Willcocks et al. (2008) have found that low copy number of *Fc gamma receptor 3B* (*FCGR3B*), which is correlated with protein expression and immune complex uptake, was associated with SLE, implying that the association of this gene CNVs with SLE may influence protein expression and function and further confer risk for the predisposition of AIDs. Molokhia et al. (2011) identified that low copy number of *FCGR3B* was associated with the risk of SLE in the Afro-Caribbean population, but not in the African ancestry population. Chen et al. (2014) further showed that low copy numbers (less than two copies) of *FCGR3A* or *FCGR3B* were significantly associated with SLE, and high copy numbers (more than two copies) of *FCGR3A* were also related to SLE onset in the Taiwanese population. Another team, Barbosa et al. (2018), detected CNV at whole-genome level using a case–control design and showed that increased FCGR3B/ADAM3A copy number was a protective factor against SLE development. In addition, they, for the first time, uncovered heterozygous deletions overlapping the CFHR4, CFHR5, and HLA-DPB2 genes in SLE patients. Notably, different genetic manifestations attributing to different backgrounds may present different trends of gene CNV association with diseases.

Heat shock proteins 90 (HSP90) is a pivotal modulator of multiple innate and adaptive inflammatory processes (Tamura et al., 2012). HSP90 has two major cytosolic isoforms, HSP90AA1 and HSP90AB1. Zhang et al. (2019) showed that HSP90AB1 CNV was correlated with SLE in the Chinese Han population, especially in females, implying that HSP90AB1 CNV is involved in the pathomechanism and development of SLE.

## Rheumatoid Arthritis

Rheumatoid arthritis (RA) is characterized by a massive tissue infiltration of inflammatory cells and affects approximately 1% of the adult population worldwide. Clinically, it mainly causes the chronic inflammation of synovial joints, which will result in the progressive destruction of the cartilage and bone (Achour et al., 2018). Many lines of data have implied the association of RA with genetic variations including SNPs and CNVs of several immune-related genes (McKinney et al., 2008; Graf et al., 2012; Olsson et al., 2012).

It is intriguing whether *FCGR3B* CNV is involved in RA. Graf et al. (2012) found a significant association between low *FCGR3B* copy number and RA (Rahbari et al., 2017). Chen et al. (2014) revealed a significant association of RA with low copy number of *FCGR3A*, but not *FCGR3B* in a Chinese cohort. Then, Rahbari et al. (2017) verified that RA patients from the UK had decreased copy number of *FCGR3B*. More recently, Ben Kilani et al. (2019) reported that genotypes without null allele of *FCGR3B* gene (copy numbers range from 1 to 3) were significantly associated with RA. In addition, increased *FCGR3B* copy number was only found in RA, and deletion of *FCGR3B* may have a protective effect on RA. The discrepancies on the correlation of RA with *FCGR3B* CNVs may partly be due to different genetic backgrounds, and this requires further investigation in different ethnic populations.

CNVs of *CCL3L1* are potentially associated with RA. McKinney et al. (2008) uncovered that a higher than two copy number of *CCL3L1* gene was a risk factor for RA in the New Zealand population (Wouters et al., 2009). Mohamed et al. showed that *CCL3L1* copy number varies from 0 to 4 in the French population and from 0 to 7 in the Tunisian population. In addition, five copies of *CCL3L1* gene protect people from RA development in the Tunisian population (Ben Kilani et al., 2016). The results showing ethnic heterogeneity of *CCL3L1* among different populations suggest that *CCL3L1* is a susceptible factor for RA, and these differences of results may be due to genetic background and/or environmental differences existing among various populations and the different technical detection methods for genotyping multi-allelic CNVs. In the future, more reliable technologies are needed to explore the relationship between *CCL3L1* CNVs and RA in more populations to identify the results.

Inflammation is the central trait of RA, accompanied with the production of reactive oxygen species (ROS), which induces oxidative damages to cellular molecules including DNA and lipids, causing diverse cytotoxic products (Fahmi et al., 2002). The *neutrophil cytosolic factor 1* (*NCF1*) gene encodes one of five sub-units of the NADPH oxidase (NOX2) complex, which could produce ROS in the immune cells, including antigen presenting cells (APCs), phagocytes, etc. Olsson et al. (2012) found that RA patients are less likely to have an elevated copy number of *NCF1* compared to controls, which may suggest that a higher copy number of *NCF1* could be a protective factor for RA. Glutathione S-transferase (GST), a multifunctional enzyme that is required for the detoxification mechanism against ROS products, exerts a vital protective antioxidant function in cells against ROS aggression (Blackburn et al., 2006). In 2011, a Swedish study found that more than one copy of *glutathione S-transferase M1* (*GSTM1*), a member of the GST family, seems to be a risk factor for autoantibody-positive RA in non-smoking females of age older than 60 years, and GSTM1 acts as a protective factor in ACPA-negative smoking men, suggesting that the copy number of the *GSTM1* gene is correlated with the development and severity of RA (Lundström et al., 2011). In 2016, it was also found that *GSTM1* is deleted in Tunisian anti-cyclic citrullinated peptide (anti-CCP)-positive RA patients, although a genetic association of *GSTM1* CNV with predisposition to RA was not detected (Achour et al., 2018). The above data indicate that *GSTM1* CNVs do not influence the susceptibility to RA, but may have an effect on its severity because deletion of *GSTM1* could increase the risk of anti-CCP-positive RA. These interesting results showed that the CNVs of inflammatory-related genes may be involved in the development of RA through influencing the production of ROS and oxidative damages to cellular molecules.

## Inflammatory Bowel Disease

Inflammatory bowel disease (IBD) is a kind of organ-specific inflammatory disease and has two main clinical subtypes, ulcerative colitis (UC) and Crohn’s disease (CD). The occurrence of CD is 300 per 100,000 people in the population with European ancestry and increasing in other ethnic population. CD affects the gastrointestinal tract and has such symptoms, like diarrhea, abdominal pain, and aberrant weight loss (Cleynen et al., 2016). Studies have demonstrated the associations between *C4* gene and several AIDs (Hauptmann et al., 1974; Hou et al., 2013), and the team led by Cleynen et al. (2016) showed that CD cases tended to have lower *C4L* and higher *C4S* copies. They also found that serum C4 protein level was not significantly different between CD patients and controls, but CD patients with higher *C4* copy number may have higher serum C4 concentration (Cleynen et al., 2016). These results suggest that more *C4* copy number may lead to higher *C4* expression and there may be a dose–efficiency correlation between *C4* copy number and protein expression in CD patients. Asano et al. (2013) studied polymorphisms of *FCGR* genes in the Japanese population and found that *FCGR3B* copy number was related to susceptibility of UC.

Defensins are endogenous antimicrobial peptides to protect the intestinal mucosa against bacterial invasion. Fellermann et al. (2006) showed that healthy volunteers as well as UC patients have 2 to 10 copies of the *human beta-defensin 2* (*HBD-2*) gene with the median of 4 copies. However, patients with colonic CD have lower *HBD-2* copy than the controls. In addition, they also found that less than four copies of *HBD-2* gene were correlated with diminished mucosal HBD-2 mRNA expression (Fellermann et al., 2006). The *DEFA1A3* gene encodes alpha-defensins 1–3. Jespersgaard et al. (2011) found that a higher *DEFA1A3* copy number was related to CD, especially to colonic CD.

## Psoriasis

Psoriasis is a serious inflammatory disease of the skin, scalp, nails, and joints and has a prevalence of about 2% in the populations of developed countries (McKinney et al., 2008). Multiple studies have identified a strong genetic component in the development of psoriasis and demonstrated the relationship between CNV of some genes and psoriasis. Wu et al. (2014) found that Chinese patients with psoriasis vulgaris had a higher copy number (more than two copies) of *FCGR3B* compared to controls through a case–control study. Carpenter et al. (2011) found no association between *CCL3L1* copy number and psoriasis. Hollox et al. studied psoriasis in Dutch and German populations and found significant associations between higher genomic copy number for beta-defensin genes (DEFB4, SPAG11, DEFB103, DEFB104, DEFB105, DEFB106, and DEFB107) and the risk of psoriasis in both of these cohorts.

IL-22, which belongs to IL-10 cytokine family, has a significant proliferative effect on different cell lines and a role of immune regulation. Prans et al. (2013) showed that the copy number variation in exon 1 but not exon 5 of IL-22 gene was significantly correlated with the severity of psoriasis.

With the emergence of new technologies, more loci with CNVs have been identified to be associated with psoriasis. Bergboer et al. (2012) utilized a pooling approach, genome-wide CNV analysis, and array comparative genomic hybridization to detect CNV variability in psoriasis and found that the absence of the late cornified envelope (*LCE*) gene cluster members *LCE3B* and *LCE3C* (LCE3C-LCE3B-del) was significantly associated with the predisposition of psoriasis in populations from Netherlands.

## Behcet’s Disease

Behcet’s disease (BD) is an immune-mediated systemic inflammatory disorder involving non-granulomatous uveitis, recurrent oral and genital ulcers, as well as skin lesions (Yang et al., 2008a). It has been verified to be associated with the *HLA-B51* gene (Xu et al., 2015). Further studies have provided new insight into the pathogenesis of BD and attracted more attentions on whether complement is involved in BD. Hou et al. (2013) investigated the relationship of CNV of *C4A* and *C4B* with BD and found that the frequency of more than two copies of *C4A* was significantly increased in BD patients, and *C4A* CNV was an independent risk factor for BD. Moreover, *C4A* expression was significantly increased in BD patients with high *C4A* copy number than with low *C4A* copy number (Hou et al., 2013). Xu et al. (2015) found that BD patients have increased frequencies of more than two *C3* copies, and C5 CNV was associated with BD. Furthermore, interleukin-17 (IL-17) and IFN-gamma expressions were upregulated in BD patients with high *C3* copy number, but not in BD patients with high *C5* copy number (Xu et al., 2015). The results imply that there are indeed CNVs in complement-related genes, and the CNVs in these genes may be involved in the development of BD.

There were also some studies targeting the relationship between *FCGR3B* CNVs and BD. Black et al. (2012) found that CNV of the *FCGR3B* gene was associated with the risk of BD in the Iranian population. The risk of BD was decreased by 40% in people with less than two copies of *FCGR3B* and by 25% in people with more than two copies of *FCGR3B*, although these tendencies were not statistically significant. They concluded that no association exists between high or low copy number of *FCGR3B* and BD or its clinical features (Black et al., 2012). Of course, further studies are need to identify this result in other populations.

Toll-like receptors (TLRs), along with RIG-I-like receptor and NOD-like receptors, belong to the family of pattern recognition receptors and contain 10 functional members in human beings, namely, TLR1–10 (Medzhitov et al., 1997). TLRs have been known to play important functional roles in several AIDs and inflammatory disorders (Chang et al., 2004; Chang et al., 2006). Fang et al. (2015) uncovered that more than 98% of people tested have two copies of all *TLRs* except for *TLR7*. In addition, they found that compared to healthy controls, male BD patients had an increased frequency of more than one copy of TLR7, and female BD patients had an increased frequency of more than two copies of this gene. This research suggested that a high *TLR7* copy number may contribute to the pathogenesis of BD (Fang et al., 2015). Therefore, from this research, we included that *TLRs* also play a potential role in BD, and the mechanism underlying this still needs to be clarified.

Many interleukins play vital roles in BD development. Hou et al. (2015) showed that BD patients have increased frequencies of more than two copies of *IL-17F* and *IL-23A*, and after stratified by sex, the association just exists in male BD patients. IL-17F protein expression is positively correlated with its gene copy number, and higher *IL-17F* copies are associated with enhanced proliferation of peripheral blood mononuclear cells (PBMC) (Hou et al., 2015). This suggests that not only SNPs but also CNVs of interleukins are involved in the pathogenesis of BD. Liao et al. (2015) investigated the association of some transcript factors with BD and showed that high CNV of related orphan receptor (*RORC*) was associated with BD susceptibility, and low *Foxp3* CNV was correlated with female BD. In addition, individuals with high *RORC* copy number seem to have relatively high mRNA levels of RORC, IL-1β, and IL-6, but not Foxp3 (Liao et al., 2015).

The disturbed apoptosis has been reported to be involved in BD development. Yu et al. (2015)investigated whether CNVs of apoptosis-related genes, including *FAS*, *caspase8*, *caspase3*, and *BCL2*, were associated with BD in the Chinese population and showed that BD patients had an increased frequency of high *FAS* copy number, and BD patients with more than two copies of *FAS* had an increased mRNA expression of *FAS* in anti-CD3/CD28 antibody-stimulated CD4^+^ T cells. Their results provided important evidence that high *FAS* copy number is involved in the pathogenesis of BD (Yu et al., 2015).

It is well-known that BD may be triggered by infectious agents in some genetically susceptible people. *DEFB4* CNV can affect the level of human beta-defensin 2, which is an inducible antimicrobial peptide. Park et al. (2011) found that *DEFB4* copy number was lower in BD samples than in controls without statistical significance, and *DEFB4* copy number was not associated with the clinical characteristics of BD. This suggests that *DEFB4* CNVs confer no risk for the susceptibility of BD (Park et al., 2011). In 2012, Ahn et al. (2012) found that 31.1% of samples had five copies of *DEFA1* with a mean of 5.4 ± 0.2. Although the distribution of *DEFA1* copy number is not different between BD patients and the controls, high *DEFA1* copy number is related to the intestinal involvement in BD, suggesting that a high *DEFA1* copy number may be associated with the development of intestinal involvement in BD (Ahn et al., 2012). Hitherto, the genes related to infectious agents may be involved in BD through changing the variation of copy number, and further researches are needed to be carried out to identify this.

There was another study that investigated the relationship between microRNA CNVs and BD, like miR-23a, miR-146a, and miR-301a. As a result, no association of CNVs of the above-mentioned miRNAs was observed in BD patients (Hou et al., 2016). However, whether other microRNA CNV is associated with BD still needs further studies to explore and clarify.

## Ankylosing Spondylitis

Ankylosing spondylitis (AS) is an inflammatory AID causing spondyloarthritis of the spine and sacroiliac joints and prevalent mainly in men with a ratio of 10:1 at the age of 20–30 years old in respect to women (Chimenti et al., 2021). Because the exact pathology of AS is still unclear, there are incoming data about the relationship between CNVs of these genes and AS, including *CCL3L1*, *FCGR3A*, *FCGR3B*, *TLR7*, *UGT2B17*, *BMP8A*, and so on. Wang et al. (2016) found that AS patients had low copies (≦3) of *FCGR3A* and *FCGR3B* in the Chinese population, implying that a lower copy number of these two genes confers risk for the susceptibility of AS. The study of Dahmani et al. (2019) found that the proportion of AS patients with less than two copies of *FCGR3A* was higher in the Algerian population and that less than two copies of *FCGR3A* was only associated with HLA-B27-negative AS patients, suggesting that *FCGR3A* deletion has an independent effect on AS regarding HLA-B27 status. Their results also showed that *CCL3L1* and *FCGR3B* CNVs may not be involved in the predisposition of AS in the Algerian population (Dahmani et al., 2019). Wang et al. (2018) found that one copy of *TLR7* was related to AS in the Chinese population after Bonferroni correction and adjustment of age and sex, and less than one copy of *TLR7* confers risk for AS susceptibility in male patients, but is a protective factor in female AS patients. Uddin et al. (2013) conducted a genome-wide CNV analysis and found that *UGT2B17* copy number was increased in a large AS multiplex family. The *UGT2B17* gene encodes an enzyme that metabolizes steroid hormones such as testosterone and selected xenobiotics (Xue et al., 2008). *UGT2B17* copy number has been shown to be related to bone mineral density and involved in the pathogenesis of osteoporosis (Yang et al., 2008b). It is known that AS in patients is often accompanied with osteoporosis (Vosse et al., 2009). This may, to some extent, explain the underlying mechanism that gain in *UGT2B17* copy number could increase the risk for AS. Bone morphogenic protein 8A (BMP8A) plays multiple functions in the formation of a bone. Shahba et al. (2018) reported that the expression of BMP8A in PBMCs is decreased in AS patients, and *BMP8A* CNVs do not influence its transcription in PBMCs and are not associated with AS susceptibility in the Iranian population. Cai et al. (2015) found that the copy number of defensing-related gene *DEFB103* was in the range of two to six in both AS patients and controls, and it was not associated with AS. More studies in different populations are needed to further identify the relationship between these gene CNVs and BD.

CD4^+^ T cells play pivotal roles in many AIDs, but whether CNVs of transcription factor genes in CD4^+^ T cells are involved in AS remains poorly defined. Bai et al. (2016) investigated whether CNV of transcription factor genes in CD4^+^ T cells including T-bet, GATA binding protein 3 (*GATA-3*), *RORC*, and fork-head box protein 3 (*FOXP3*) are associated with acute anterior uveitis (AAU) in the presence or absence of AS. They found that a higher *T-bet* copy number is more common in AAU+, AS+ and AAU+, AS− cases compared with healthy controls. Additionally, the frequency of AAU+, AS+ patients with high *GATA-3* copy is higher, and the proportion of female AAU+, AS+ patients with high *FOXP3* copy number is also higher than that of other populations, but the copy number of *RORC* is not correlated with AAU+, AS+ or AAU+, AS− patients (Bai et al., 2016).

People are also interested in the relationship between CNVs of various microRNAs (miRNAs). Yang et al. (2017) studied the association between CNVs of miRNAs and AS and found that the frequencies of AAU+, AS+ patients with low copy numbers of miR-143, miR-146a, miR-9-3, and miR-205 as well as high copy numbers of miR-301a and miR-23a all increased, and the frequencies of patients with AAU+, AS− with low copy number of miR-146a and high copy numbers of miR-23a and miR-205 are significantly different. In addition, they found that miR-9-3 mRNA expression is significantly decreased in AAU+, AS+ patients and positively correlated with its copy number (Yang et al., 2017).

## Primary Sjogren’s Syndrome

Primary Sjogren’s syndrome (pSS) is characterized by the presence of circulating autoantibody (anti-Ro/SSA and anti-La/SSB), as well as the involvement of the exocrine glands (salivary and lacrimal gland), joint, and muscle (Nossent et al., 2012; Haldorsen et al., 2013). There were few studies on the issue of *FCGR3B* CNVs and *pSS.* Mamtani et al. (2010) showed that the median *FCGR3B* gene copy is two in the cohort of Spanish ancestry. The risk of pSS would increase if people carry less or more than two copies of *FCGR3B* (Mamtani et al., 2010). Nossent et al. (2012) conducted a case–control study and found that less than two copies of *FCGR3B* can confer risk for pSS in the Australian population, and low FCGR3B copy number is associated with the levels of rheumatoid factor (RF) titers and serum IgG, but not with anti-Ro ± La autoantibodies. They further identified that *FCGR3R* CNV is a genetic susceptibility factor for pSS (Liao et al., 2015). However, Haldorsen et al. (2013) showed no association of *FCGR3B* CNV with pSS in the Norway and Switzerland populations. To clarify, these controversial results need more studies in more populations with different ethnicities and regions.

## Type 1 Diabetes Mellitus

Type 1 diabetes mellitus (T1DM) is characterized by β cell destruction in the pancreas and the production of antibodies against β cells, with a high prevalence of 1 in 350 teenagers in the UK (Bluestone et al., 2010). There is a series of symptoms in T1DM, such as polydipsia, polyuria, polyphagia, and weight loss. *C4* is a gene of the highly variable complement pathway situated ∼500 kb from DRB1 and DQB1 and strongly associated with diverse AIDs (Hauptmann et al., 1974; Hou et al., 2013; Cleynen et al., 2016), so some scientists also carried out numerous researches to uncover whether *C4* is associated with T1DM development. Kingery et al. (2012) found that higher *C4A* copy number tends to be correlated with the protection of residual β-cell function in new-onset T1DM patients, while lower *C4B* copy number is related to the end of disease remission at 9 months post diagnosis. Mason et al. (2014) explored the relationship between *C4* CNV and T1DM and found that individuals with T1DM have significantly fewer copies of HERV-K (C4), one notable component of *C4*. About the relationship between *CCL3L1*, people also did a lot of work. McKinney et al. (2008) revealed an association between *CCL3L1* CNVs and T1DM in the Caucasian population from New Zealand, but the association was not statistically significant with *P* of 0.064. Then, the Wellcome Trust Case Control Consortium found no association between *CCL3L1* CNV and T1DM (Wellcome Trust Case Control Consortium et al., 2010). These work are consistent and implies that *CCL3L1* CNVs may be not associated with T1DM. *FCGR3B* CNVs are involved in the pathogenesis of several AIDs, such as SLE and RA, because its role in the clearance of immune complexes is impaired in these disease settings (Willcocks et al., 2008; Graf et al., 2012). Almal and Padh (2015) found that *FCGR3B* copy number in the Indian population varies significantly from zero to two per diploid genome in other populations, which helps us to understand the potential role of *FCGR3B* CNV and its association with AIDs in the Indian population.

Killer immunoglobulin-like receptors (KIRs) reside on the surface of natural killer cells to bind to their corresponding human leukocyte antigen (HLA) class I ligands. It is noted that KIRs are vital candidates for HLA-associated AIDs, including T1DM. Although Pontikos et al. (2014) did not find a relation of *KIR3DL1/3DS1* copy number to T1DM in the white European population, Grayson et al. (2010) utilized a more powerful genome-wide CNV analysis and found 39 CNVs either enriched or depleted in T1DM patients, including a deletion on chromosome 6p21, near an HLA-DQ allele. Their results indicated that both enrichment and depletion of these genes are high risk factors for developing T1DM, and genetic variants such as CNVs may contribute to the development of islet autoimmunity in T1DM (Grayson et al., 2010).

## Autoimmune Thyroid Disease

Autoimmune thyroid disease (AITD), which mainly includes Graves’ disease (GD), Hashimoto’s thyroiditis (HT), and Graves’ ophthalmopathy (GO), is a kind of organ-specific AID with a prevalence of 5% of the overall population (Jin et al., 2018). GD is characterized by hyperthyroidism caused by positive autoantibodies against thyroid-stimulating hormone receptors (TSHR), and HT is often characterized by positive anti-thyroid peroxidase antibody (TPOAb), anti-thyroglobulin antibody (TgAb), and hypothyroidism. There have been many studies focusing upon the relationship between the CNVs of immune-related genes and the development of AITD, and lots of interesting results have been reported. The two earliest studies on CNVs and AITD were both published in 2011. One study aimed to explore the association between CNVs of immune regulatory genes and AITD and found no CNVs of *CD40* and *CTLA4* genes in GD cases and few *PTPN22* CNVs in two GD individuals (Huber et al., 2011). Liu et al. (2011) found that CNVs of *C4*, *C4A*, and *C4B* contribute to the predisposition of GD, but not GO. Liao et al. (2014) revealed that *TSHR* CNVs harbor the etiology of GD, but not GO. Besides, they also observed that only female GD patients have fewer *TLR7* copies, and there is no significant association between sex and *TLR7* CNVs (Liao et al., 2014). GD is a disease predominantly in females (Manji et al., 2006); therefore, they believed that *TLR7* CNVs affect the pathogenesis of female GD due to the gender-dependent immune response (Liao et al., 2014). In 2017, we conducted microarray to explore the profile of genes with CNVs in GD and found some genes with copy number gain. In addition, seven of these genes including *CFH*, *CFHR1*, *KIAA0125*, *UGT2B15*, *UGT2B17*, *TRY6*, and *CCL3L1* were chosen to further validate these findings in an expanded cohort. The results showed no correlation between CNVs of these genes and GD (Song et al., 2017). Jin et al. (2018) assessed CNVs of two immune-related genes *SIRPB1* and *TMEM91* in AITD and found that the distributions of *SIRPB1* copy number were different between AITD patients and the controls, implying *SIRPB1* is a risk factor for AITD. Guan et al. (2020) showed that the distribution of copy numbers of cell growth-related genes glypican-5 (*GPC5*), B9 domain-containing 2 (*B9D2*), as well as ankyrin repeat and suppressor of cytokine signaling (SOCS) box-containing protein 11 (*ASB11*) is different in AITD patients and the controls, and *GPC5* CNVs are risk factors for AITD. However, they did not find any association between their CNVs and the occurrence of AITD (Guan et al., 2020). The different relationship between CNVs of these genes and different sub-types of AITD implies the diverse genetic mechanisms underlying AITD. Overall, researchers have found that CNVs of several thyroid-susceptible genes are correlated with the development of AITD. Studies on the correlation between CNVs and AITD susceptibility inevitably deepen our understanding of the pathogenetic mechanism of AITD and further promote molecular diagnosis and therapies of AITD. These studies were mainly done in Chinese population; in the future, we will need further studies in more populations to identify these results.

## Future Outlook

To further gain new and deep insights on the genetic mechanism of AIDs, we reviewed the association between different AIDs and CNVs of some genes with potential pivotal roles in the development of AIDs. This review provides an update evolving the view of copy number variations in AIDs. For the first, several CNVs are very common in diverse AIDs, implying these AIDs may potentially possess a similar genetic pathomechanism. Therefore, more association studies should be done on some other diseases when a certain link is identified between some CNVs and a specific AID. For the second, some CNVs are differently distributed in various diseases in different ethnic populations, suggesting that AIDs have their own different phenotypes and different genetic and/or environmental backgrounds among diverse populations. Herein, more researches aiming to uncover the relationship between environmental factors and diseases and the influences of environmental elements on immunity should be encouraged. For the third, with the continuous advancement in genotyping technology such as the high-throughput whole-genome sequencing method, more susceptible variants will be found. Thus, further replication studies should be conducted to confirm the results of studies with different ethnic cohorts and independent populations.

For that, CNVs may be an important pathogenesis of AIDs; CNVs will also become an effective way to study the molecular mechanism of AIDs; and we can find molecular markers for genetic diagnosis or judgment of prognosis of this kind of disease. At the same time, it opens a new page for the research of AIDs and is becoming a new research hotspot. We believe that genetic diagnosis or judgment of prognosis based on CNVs will cover more AID spectrum and benefit a wider population. It is believed that with the high-throughput genome-wide CNV scanning platform and the new development of statistical calculation method, GWAS based on CNVs, a new genetic susceptibility marker, will become a powerful tool to study the genetic susceptibility of AIDs, just like the traditional GWAS based on SNPs and their haplotypes. These two complementary genetic markers will help us to understand the molecular mechanism, identify susceptible genes, and understand the relationship between genetic variations and disease phenotype of complex diseases, such as AIDs, which is of great significance. Simultaneously, to our hope, more functional experiments and more replication studies should be done and collected, and an entire autoimmune CNVs database should be set up, which can be searched easily and help us to understand the pathogenesis of AIDs much better. GWAS has already pointed toward genetic susceptibility loci and potential mechanisms of pathogenicity. Chromosomal microarrays have added little further information. It therefore seems unlikely that whole-genome sequencing alone will answer the necessary questions. Rather, genomic DNA approaches will likely need to be combined with other measures, such as RNA sequencing and/or proteomics to solve many of the remaining questions at hand.

Although CNVs describe the pathogenesis of AIDs from a new perspective, it is still early to explain the occurrence and development of complex diseases only by CNVs at the genomic level because the molecular mechanism of complex diseases at the chromosomal level is not completely clear. Due to the existence of multiple mechanisms of CNVs and the different effects of CNVs on molecular phenotype and gene expression, therefore, the interpretation of clinical significance and genetic mode of CNVs must be done more carefully, and it should be based on the comprehensive assessment of genomic variation.
